# Investigating the Mitochondrial Permeability Transition Pore in Disease Phenotypes and Drug Screening

**DOI:** 10.1002/cpph.59

**Published:** 2019-05-13

**Authors:** Gauri Bhosale, Michael R. Duchen

**Affiliations:** ^1^ UCL Consortium for Mitochondrial Research and Department of Cell and Developmental Biology University College London London United Kingdom

**Keywords:** calcium, mitochondria, permeability transition pore

## Abstract

Mitochondria act as ‘sinks’ for Ca^2+^ signaling, with mitochondrial Ca^2+^ uptake linking physiological stimuli to increased ATP production. However, mitochondrial Ca^2+^ overload can induce a cellular catastrophe by opening of the mitochondrial permeability transition pore (mPTP). This pore is a large conductance pathway in the inner mitochondrial membrane that causes bioenergetic collapse and appears to represent a final common path to cell death in many diseases. The role of the mPTP as a determinant of disease outcome is best established in ischemia/reperfusion injury in the heart, brain, and kidney, and it is also implicated in neurodegenerative disorders and muscular dystrophies. As the probability of pore opening can be modulated by drugs, it represents a useful pharmacological target for translational research in drug discovery. Described in this unit is a protocol utilizing isolated mitochondria to quantify this phenomenon and to develop a high‐throughput platform for phenotypic screens for Ca^2+^ dyshomeostasis. © 2019 The Authors. This is an open access article under the terms of the Creative Commons Attribution License, which permits use, distribution and reproduction in any medium, provided the original work is properly cited.

## INTRODUCTION

As a universal second messenger, Ca^2+^ plays a critical role in a wide range of cellular processes. At rest, intracellular [Ca^2+^] gradients are tightly regulated; whereas extracellular [Ca^2+^] is at ∼1 mM, cytosolic [Ca^2+^] ([Ca^2+^]_c_) and mitochondrial [Ca^2+^] ([Ca^2+^]_m_) are maintained at close to 100 nM. This unique electrochemical gradient allows the low [Ca^2+^]_c_ to undergo a substantial proportional increase, with a small and therefore energetically inexpensive absolute change, in response to a stimulus. Such changes in [Ca^2+^]_c_ are a fundamental aspect of cell physiology in all tissues, underlying excitation contraction coupling, secretion, and motility (Denton & McCormack, 1985; Duchen, 1992; Duchen, Leyssens, & Crompton, 1998; Prudent et al., 2016).

Ca^2+^ signaling is subverted in multiple pathologies to drive cell death through opening of the mitochondrial permeability transition pore (mPTP). Although opening of the pore is triggered primarily by an increase in mitochondrial Ca^2+^ load, it can also be induced by oxidative stress. Pore opening leads to enhanced permeability of the inner mitochondrial membrane (IMM) to solutes of ≤1.5 kDa, resulting in membrane rupture, bioenergetic failure, and ultimately cell death. Seminal studies by Crompton and colleagues in the 1980s revealed that pore opening could be inhibited by cyclosporin A (CsA), suggesting that the mPTP represents a viable drug target (Crompton, Ellinger, & Costi, 1988). The association of this phenomenon with disease has resulted in attempts to develop targeted therapies using screening assays to identify compounds that affect mPTP function (Briston, Selwood, Szabadkai, & Duchen, 2019). Although cell‐permeant fluorescent dyes can assist in capturing mPTP opening in an intact cell, the results obtained can be variable, as protocols both to induce and to inhibit mPTP opening have proven to be unreliable. mPTP opening has long been studied in isolated mitochondria through very robust protocols that rely on measurements of light scattering or fluorescence measurements following bolus additions of calcium.

In this unit, mitochondrial Ca^2+^ buffering is measured in isolated mitochondria (Basic Protocol 1) using a Ca^2+^‐sensing fluorescent extra‐mitochondrial dye (Basic Protocol 2). Often, healthy mitochondrial function is pivotal to Ca^2+^ uptake, and therefore, it is crucial to ensure that the mitochondria are not damaged during isolation.

**Figure 1:**
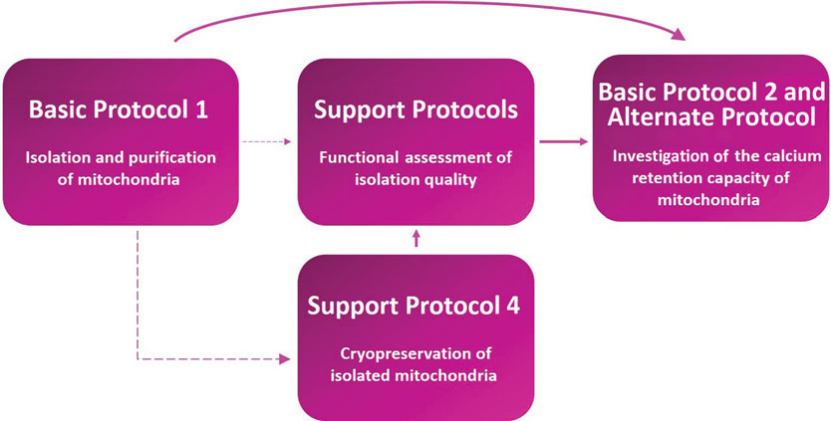
Experimental workflow depicting the order in which each protocol, whether basic or support, needs to be executed. The solid lines indicate pathways that are necessary, whereas the dashed lines indicate pathways that are optional. The information needed to determine the appropriate sequence of experiments is specified in further detail in the text.

This unit details several protocols to allow the user to assay the mPTP in isolated mitochondria. Basic Protocol 1 involves isolation and purification of mitochondria from a biological sample using differential centrifugation to obtain functional mitochondria that can buffer Ca^2+^. Mitochondrial quality and functional integrity can be assessed using support protocols for the following: measurement of mitochondrial membrane potential (ΔΨ_m_), which is essential for Ca^2+^ uptake, using the fluorescence indicator rhodamine‐123 (Support Protocol 1); measurement of the oxygen consumption rate (OCR) using a Clark‐type electrode (Support Protocol 2); and immunoblotting for various mitochondrial proteins as an additional surrogate for mitochondrial content and function (Support Protocol 3). These quality‐control procedures are crucial during initial optimization of the isolation technique. An additional support protocol (Support Protocol 4) details cryopreservation of mitochondria using an osmolyte, trehalose, often necessary for high‐throughput drug screens of compound libraries where large quantities of mitochondria might be needed regularly for multiple assays. Finally, Basic Protocol 2 describes a fundamental experiment to assay the capacity of mitochondria to accumulate Ca^2+^ using Ca^2+^‐sensitive fluorescent dyes and how this might vary between genotypes or treatments. The Alternate Protocol allows multiplexing of this approach with assessment of mitochondrial swelling. A workflow for the protocols is shown in Figure 1.

## Basic Protocol 1 ISOLATION AND PURIFICATION OF MITOCHONDRIA FROM MOUSE LIVER USING EXTRACTION AND CENTRIFUGATION

This protocol is used for isolating mitochondria from animal tissue, such as liver, brain, heart, or muscle. Liver from an adult mouse is specifically employed below. The key steps include tissue extraction, homogenization, and differential centrifugation.

### Materials

Adult C57/BL6J wildtype mouse (aged 3 to 6 months)
Isolation buffer (see recipe) with or without 1 mM phenylmethane sulfonyl fluoride (PMSF; 0.1 M stock in ethanol; Sigma‐Aldrich, 93482‐50ML‐F), 4°C
Dulbecco's phosphate‐buffered saline (DPBS; Gibco, 14190094), 4°C

Dissecting scissors (World Precision Instruments, 14393)
Tweezers (e.g., Style 5, Dumont, 0103‐5‐PO)
50‐ml Falcon tube (Fisher Scientific, 10788561)
100‐ml glass beaker
Scale (with ≥100 g capacity and ≥0.1 g readability error)
Tissue homogenizer (Potter‐Elvehjem Tissue Grinder with PTFE Pestle and Glass Tube, Kemble Chase, 886000‐0023)
Electric hammer drill (Black+Decker, BCD700S1K)
50‐ml Oak Ridge ultracentrifuge tubes (Thermo Scientific, 3118‐0050))
Ultracentrifuge with temperature control (Beckman Coulter J2‐MC High Speed Centrifuge) and JA‐20 fixed‐angle rotor (Beckman Coulter, 334831), 4°C
Pierce BCA Protein Assay Kit (Thermo Scientific, 23225)
Falcon 96‐well clear plastic plate (VWR, 734‐0023)
Microplate reader (e.g., Fluostar Optima, BMG Labtech)

*NOTE*: The entire procedure should be conducted on ice, using refrigerated equipment maintained at 4°C, or in a cold room.

*NOTE*: Please ensure that you follow the appropriate guidelines set by your institution, in line with national regulations, when handling animals.

**Figure 2:**
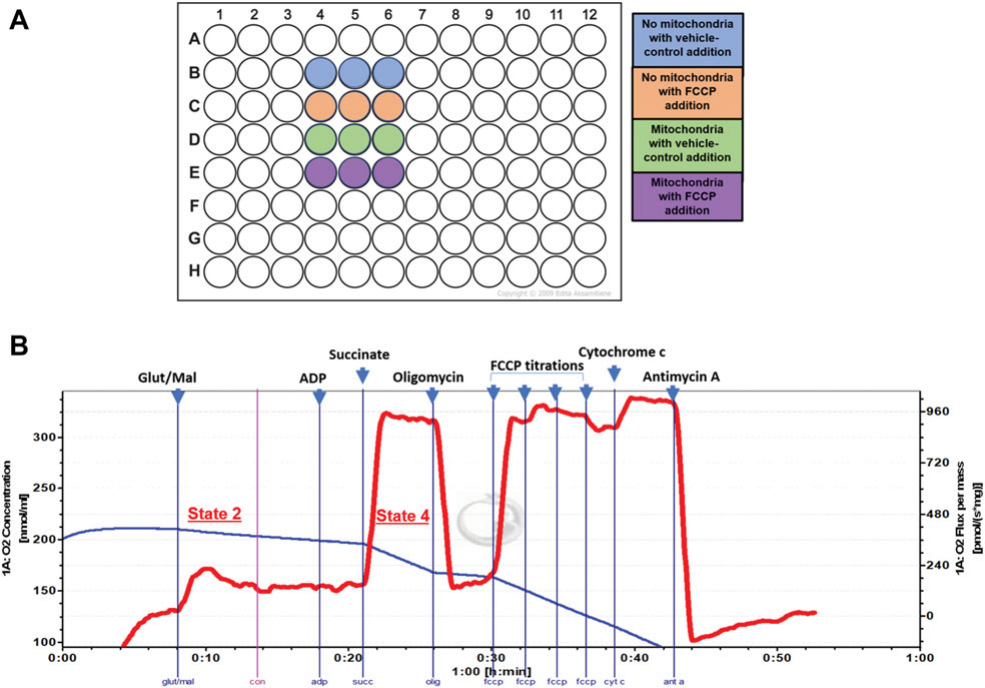
Schematic and graphical representation of Support Protocols 1 and 2. (**A**) Plate map depicting the minimum conditions required to assess ΔΨ_m_ using rhodamine‐123 in dequench mode in a fluorescence multiwell plate reader. (**B**) Oxygen consumption rate (OCR) measurements using isolated mitochondria and an Oroboros instrument. The blue line and left y‐axis depict the oxygen concentration in nmol/ml. The red line and the right y‐axis report the slope of the blue line, that is, the OCR per milligram of protein, in pmol/s/mg. Drug additions are specified in the order that they should be performed.

1 Sacrifice an adult C57/BL6J wildtype mouse using an institutionally approved procedure.
  In our procedure, we sacrifice the animal using manual cervical dislocation in compliance with the guidelines set in the Animals (Scientific Procedures) Act 1986, followed by decapitation using scissors, as this minimizes damage to the mitochondria. For most experiments, if testing ≤25 different conditions, the liver from one adult mouse (3 to 6 months of age) is sufficient.
  Alternatively, cells could be used. The number of cells needed for mitochondrial isolation will vary based on cell type. For example, the yield of mitochondria from 10 million HeLa cells or a fully confluent 10‐cm culture dish is ∼500 µg, which is enough for 10 technical replicates. We suggest isolating a sufficient amount of mitochondria for 12 replicates for a basic experiment, as shown in Figure 2A, as well as additional mitochondria for functional validation of the isolation technique.

2 Place animal on its dorsal side. Cut through abdomen using dissecting scissors and tweezers. Locate liver, between the ribcage and the gut. Dissect out entire liver, ensuring that all lobes are removed (Lampl, Crum, Davis, Milligan, & Del Gaizo Moore, 2015).
3 Place liver in 20 ml ice‐cold isolation buffer in a 50‐ml Falcon tube on ice as quickly as possible (<10 min).
4 Transfer liver from the tube to a 100‐ml glass beaker. Rinse liver in ice‐cold DPBS by pouring the DPBS on the liver and then carefully decanting it. Submerge washed tissue in DPBS and mince it with scissors into rice grain–sized pieces.
5 Repeat rinses with DPBS five times, until the blood is removed from the liver.
6 Weigh liver tissue and add isolation buffer with 1 mM PMSF, with the volume in milliliters equaling twice the tissue's weight in grams.
  For example, use 10 ml isolation buffer for 5 g tissue.
7 Transfer tissue with the isolation buffer into the glass tube of a tissue homogenizer. Macerate tissue using an electric hammer drill for 10 to 20 plunges to obtain a uniform homogenate that contains no visible pieces of tissue.
  The capacity of the glass tube as well as the corresponding pestle should be determined based on the volume of the mixture obtained in step 6.
  It is important to ensure that the strokes of the drill are slow and even.
8 Transfer homogenate to a 50‐ml Oak Ridge ultracentrifuge tube and centrifuge 10 min at 800 × *g*, 4°C, to generate a nuclear pellet.
9 Carefully transfer supernatant to another 50‐ml ultracentrifuge tube and centrifuge 10 min at 10,300 × *g*, 4°C, to isolate crude mitochondrial pellet.
  The nuclear pellet can be discarded.
10 Pipet off supernatant, carefully resuspend mitochondrial pellet in 1 ml isolation buffer with 1 mM PMSF, and place on ice.
11 Quantify protein content of the suspension using a Pierce BCA Protein Assay Kit, a Falcon 96‐well clear plastic plate, and a microplate reader.
  This step can take between 20 and 45 min.
  The Pierce BCA Protein Assay Kit allows quantification of protein content using colorimetric detection. It relies on copper‐ion reduction by proteins, with the reduced copper ions in turn reacting with bicinchoninic acid to produce the color change; this can be measured as a change in absorbance at a wavelength of 562 nm in a microplate reader. The color change follows a linear relationship with the protein concentration under the conditions described within the kit. The kit provides materials and instructions for setting up a standard curve to determine the protein concentration of your mitochondrial sample.
  Usually, a single liver can produce 4 to 10 mg/ml protein in the mitochondrial suspension from step 10. It is possible to combine the mitochondria obtained from livers from multiple animals if from the same genotype and/or treatment condition. When performing the isolation for the first time, that is, for a new source of mitochondria or a new experimental condition, functional validation of the mitochondria using Support Protocols 1 to 3 is essential before proceeding to Basic Protocol 2 or the Alternate Protocol. The mitochondrial suspension can be stored at 4°C for ≤8 hr after isolation. If mitochondria need to be preserved for longer, please refer to Support Protocol 4, which describes an alternative isolation technique for cryopreservation of functional mitochondria. A workflow for the experimental design is shown in Figure 1.

## Support Protocol 1 MEASUREMENT OF MITOCHONDRIAL MEMBRANE POTENTIAL

Healthy mitochondria maintain a negative ΔΨ_m_ across the IMM by pumping protons into the intermembrane space. This requires energy from sequential reduction of components of the electron transport chain, with oxygen as the final acceptor. This electrochemical proton gradient is essential for ATP production by ATP synthase. It is also crucial for electrogenic uptake of Ca^2+^. Therefore, this mitochondrial property can be assessed using cationic fluorescent dyes such as TMRM or rhodamine‐123. Rhodamine‐123 is used in the protocol detailed below. This protocol can be carried out immediately after Basic Protocol 1, before proceeding to Basic Protocol 2 or the Alternate Protocol.

### Materials

MSK buffer (see recipe) with 10 mM succinate (1 M stock; Sigma‐Aldrich, S3674) and 1 µM rotenone [1 mM stock in dimethyl sulfoxide (DMSO); Sigma‐Aldrich, R8875]
10 mg/ml rhodamine‐123 (Sigma‐Aldrich, R8004) in ethanol
Mitochondria (see Basic Protocol 1)
1 mM carbonyl cyanide‐p‐trifluoromethoxyphenylhydrazone (FCCP; Sigma‐Aldrich, C2920) in ethanol
190‐proof ethanol
Deionized water

Fluorescence plate reader (Fluostar Optima, BMG Labtech), including injector and two pumps
96‐well black opaque plate (VWR, 734‐1663)
37°C incubator
15‐ml Falcon tubes (Fisher Scientific, 10263041)
Excel or equivalent software

1 Set temperature of the fluorescence plate reader to 30°C.
2 Prepare 20 ml MSK buffer with 10 mM succinate and 1 µM rotenone (MSK+; see Basic Protocol 2, step 2).
3 Add 10 mg/ml rhodamine‐123 (fluorescent dye) in ethanol to a final concentration of 10 µg/ml.
  Rhodamine‐123 is used in ‘dequench’ mode. In short, the cationic dye is loaded into the mitochondria at a high concentration, causing quenching of the fluorescence, which reduces the fluorescence intensity. Upon depolarization and loss of ΔΨ_m_, the dye moves out of the mitochondria and into the buffer, where it is no longer quenched. Thus, the fluorescence signal increases in response to a loss of ΔΨ_m_ (Duchen, Surin, & Jacobson, 2003).
4 Resuspend mitochondria in the MSK+ containing rhodamine‐123 to a final concentration of 500 µg/ml. Add 45 µl mitochondrial suspension to each well of a 96‐well black opaque plate in triplicate for each condition (see Fig. 2A for an example plate design) and incubate for 30 min in a 37°C incubator.
5 Prepare two 15‐ml Falcon tubes with MSK+ buffer, with one containing 10 µM FCCP (from 1 mM stock in ethanol) and the other without FCCP (as a vehicle control).
  Five microliters of 10 µM FCCP (final concentration: 1 µM) or the vehicle control is injected at cycle 5 to depolarize the mitochondria.
6 Clean plate‐reader injector with 190‐proof ethanol and deionized water before priming the two pumps with 1 ml of the respective solutions from step 5.
7 Obtain results from samples using the plate reader once every minute until cycle 5. Continue to measure for five more cycles after addition of FCCP/vehicle control.
  The light source is a high‐energy xenon lamp. The emission from the excited sample is collected using a combination optic containing two liquid‐filled light guides for measuring fluorescence intensity. An excitation filter of 485 nm (bandpass: 12) is used, and the emitted light is detected with a side‐window photomultiplier tube after passing through a 520‐nm emission filter. Integrated injectors are used for the compound additions.
8 Collect data after completion of the five cycles and plot fluorescence intensities against time using Excel or equivalent software.
  Addition of FCCP should cause an increase in fluorescence intensity measurements due to depolarization, indicating an intact ΔΨ_m_ at the start. This increase should be absent from the wells with no mitochondria and those with the vehicle (negative) control added.

## Support Protocol 2 MEASUREMENT OF OXYGEN CONSUMPTION RATE

Healthy coupled mitochondria utilize oxygen at complex IV of the electron transport chain to enable ATP production. Therefore, in the presence of appropriate substrates, an oxygen electrode can be employed to measure the OCR to gauge the health of mitochondria. An Oroboros Power O2k‐Respirometer is used in the protocol below; this instrument contains two chambers where dissolved oxygen is measured by a Clark‐type polarographic oxygen electrode as an amperometric signal that is in turn converted into a voltage. The data obtained from this protocol provide an estimate of the efficiency of oxidative phosphorylation within the isolated mitochondria (Basic Protocol 1), a parameter that should remain consistent across different isolations. This protocol can be used in conjunction with Support Protocol 1 to ascertain the viability of the mitochondria obtained from Basic Protocol 1.

### Materials

Miro5 buffer (see recipe)
Mitochondria (see Basic Protocol 1)
2 M glutamate (Sigma, G1251)
800 mM malate (Sigma‐Aldrich, M1000)
1 M succinate (Sigma‐Aldrich, S3674)
1 mM rotenone (Sigma‐Aldrich, R8875) in DMSO
500 mM adenosine diphosphate (ADP; Sigma, 01905)
5 mM cytochrome c (Sigma‐Aldrich, C3131)
5 mM oligomycin complex (Sigma‐Aldrich, O4876) in DMSO
1 mM FCCP (Sigma‐Aldrich, C2920) in ethanol
5 mM antimycin A (Sigma‐Aldrich, A8674) in ethanol

Power O2k‐Respirometer (for high‐resolution respirometry; Oroboros Instruments, 10023‐02), including sensors, Clark‐type electrode chambers with stoppers, and DatLab software
Hamilton syringes (10 µl and 25 µl)

1 Turn on Power O2k‐Respirometer. Calibrate sensors in the Clarke‐type electrode chambers using 2 ml Miro5 buffer (respiration medium) to determine the oxygen saturation at 37°C (see manufacturer's instructions), with stirring at 750 rpm.
  The temperature and stirring can be set using the DatLab software controlling the respirometer.
2 Add 100 to 300 µg mitochondria to each chamber and close stoppers to create a closed O_2_ system.
  The amount of mitochondria used for respirometry measurements must be optimized. In general, ensure that the basal OCR is >20 pmol per second per milliliter per milligram of protein. Furthermore, the OCR should not be so high as to cause anoxia during the course of the experiment. Please refer to Figure 2B for an example trace to complement the following steps.
3 Once the OCR stabilizes (see red line in Fig. 2B; this should take 5 to 10 min), using Hamilton syringes, add 2 M glutamate (final concentration, 10 mM) and 800 mM malate (final concentration, 2 mM).
  This will achieve State 2, or complex I–linked respiration, which is the OCR due to the flow of electrons from complex I as glutamate and malate provide electrons to complex I.
  Use Hamilton syringes for compound additions in all steps.
4 Add 1 M succinate (final concentration, 10 mM) and 1 mM rotenone in DMSO (final concentration, 1 µM) to chambers.
  This will result in complex II–linked respiration, as rotenone inhibits complex I and succinate bypasses this inhibition and provides electrons to complex II.
5 After the OCR stabilizes, add 500 mM ADP (final concentration, 5 mM) to chambers.
  The stable OCR after the addition of ADP provides a measure of State 4. This is the maximal OCR in the presence of a saturating ADP concentration; that is, State 4 is the maximum OCR when ADP phosphorylation is coupled with oxygen consumption.
6 Add 5 mM cytochrome c (final concentration, 10 µM) to the respiring mitochondria.
  The integrity of the outer mitochondrial membrane (OMM) is tested by observing the effect of cytochrome c addition on the OCR and comparing this OCR to State 4 respiration. An increase of <10% indicates an intact OMM. This is an important step in the functional validation of the mitochondrial isolation technique.
7 Add 5 mM oligomycin complex in DMSO (final concentration, 5 µM).
  This step inhibits ATP synthase, thereby identifying the OCR required for ATP production. A substantial decrease in the OCR indicates an intact IMM, with minimal leakage of protons across this barrier, and identifies the rate of oxygen consumption required to sustain ATP turnover.
8 Titrate FCCP (sequential additions of 0.5 µM from a stock of 1 mM FCCP in ethanol) until maximal uncoupled respiration is attained. Add 5 mM antimycin A, a complex III inhibitor, in ethanol to a final concentration of 2.5 µM to determine the non‐mitochondrial OCR.
9 Analyze data using the DatLab software after acquisition.
  In brief, the average value of the OCR (see red line in Fig. 2B) after stabilization following drug addition can be extracted, and thus, the effect of the drugs can be compared across different isolations.
  These assay procedures should be conducted routinely to ensure consistent and accurate mitochondrial isolation, such as every 3 to 5 isolations. A representative trace with expected values is shown in Figure 2B. In particular, ΔΨ_m_ and the State 4 OCR should be reproducible across the preparations from the same mitochondrial source on different days, that is, within a 10% error margin.

## Support Protocol 3 IMMUNOBLOTTING FOR MITOCHONDRIAL PROTEINS

This protocol summarizes a procedure to quantify mitochondrial proteins using immunoblotting, thus validating the mitochondrial isolation (Basic Protocol 1) by probing for mitochondrially enriched proteins. As loss of cytochrome c from the mitochondria indicates a ruptured OMM, it is possible to diagnose problems with isolation by quantifying the levels of cytochrome c retained in the mitochondrial pellet. Other proteins that can be investigated by immunoblotting include TOM20, VDAC (OMM), subunits of the components of the electron transport chain, and subunits of ATP synthase. Shown below are a few key steps for western blotting; this procedure can be adapted for different equipment and reagents.

### Materials

Mitochondria (see Basic Protocol 1)
NuPAGE LDS Sample Buffer (4×; Invitrogen, NP0008) with 5% (v/v) β‐mercaptoethanol (Sigma, M6250)
Protein standards (Precision Plus Protein Kaleidoscope Prestained Protein Standards, Bio‐Rad, 1610375)
NuPAGE 4‐12% Bis‐Tris Protein Gel (Invitrogen, NP0321BOX)
NuPAGE MOPS SDS Running Buffer (Invitrogen, NP0001)
Immobilon‐P PVDF membrane (Millipore, IPVH00010)
Methanol
NuPAGE Transfer Buffer (Invitrogen, NP0006) with 20% (v/v) methanol
5% (w/v) skim milk (Millipore, 1153630500) in TBS‐T (see below)
Primary antibody (recommended: anti–cytochrome c, Abcam, ab13575; anti‐VDAC1, Abcam, ab15895; anti‐TOM20, Santa Cruz Biotechnology, sc‐136211; Total OXPHOS Rodent WB Antibody Cocktail, Abcam, ab110413; ensure that species origin of antibody is compatible with experimental setup)
Tris‐buffered saline (TBS; see recipe) with 0.1% (v/v) Tween‐20 (Sigma, P9416) (TBS‐T)
Horseradish peroxidase (HRP)–conjugated IgG secondary antibody (Sigma, A9044 or A0545)
Chemiluminescent reagent (e.g., Luminata Forte Western HRP substrate, Merck Millipore, WBLUF0100)

1.5‐ml Eppendorf tube
50°C heating block
Protein gel electrophoresis chamber system (XCell SureLock Mini‐Cell, Thermo Fisher, EI0001)
Power supply (e.g., PowerPac HC High‐Current Power Supply, Bio‐Rad, 1645052)
Semi‐dry blot transfer system (Novex Semi‐Dry Blotter, Invitrogen, SD1000)
Blot imaging system (ChemiDoc XRS^+^ System with Image Lab Software, Bio‐Rad, 1708265)
ImageJ2 software (National Institutes of Health)

1 For each sample, heat 50 μg mitochondria in NuPAGE LDS Sample Buffer with 5% β‐mercaptoethanol in a 1.5‐ml Eppendorf tube for 5 min in a 50°C heating block for each sample.
  The sample buffer is supplied as a 4× stock, and the volume needed will be determined by the volume of mitochondrial suspension used.
  Critically, a lower temperature is used to preserve the structure of target epitopes of the mitochondrial proteins.
2 Load samples and protein standards into the wells of a NuPAGE 4‐12% Bis‐Tris Protein Gel immersed in NuPAGE MOPS SDS Running Buffer and separate proteins using a protein gel electrophoresis chamber system and a power supply according to the manufacturers’ instructions.
  Ideally, the gel is run at 150 V for 45 min (the dye front should have run 80% down the gel) before moving on to step 3. The same mitochondrial sample can be run in multiple wells so that multiple primary antibodies can be used to probe for different proteins. The user should probe for cytochrome c at a minimum as a measure of an intact OMM in the mitochondria in the suspension.
3 Activate an Immobilon‐P PVDF membrane with methanol for 1 min. Transfer proteins from the gel to the PVDF membrane in NuPAGE Transfer Buffer with 20% methanol using a semi‐dry blot transfer system at 20 V for 1 hr.
4 Incubate membrane in 5% skim milk in TBS‐T for 1 hr to block the membrane.
5 Incubate membrane with the appropriate primary antibody overnight at 4°C.
6 Wash blots three times with TBS‐T for 5 min each before incubating them with the corresponding HRP‐conjugated IgG secondary antibody for 1 hr at room temperature.
7 Wash blots with TBS‐T as in step 6 and visualize protein bands using a chemiluminescent reagent and a blot imaging system.
8 Use densitometry analysis via ImageJ2 software to determine the relative expression levels of the protein across samples.
  Expression levels of the mitochondrial proteins listed above can be instrumental in functional validation of the isolation technique. In particular, one should expect sufficient detection of cytochrome c and little fluctuation between expression levels of other proteins across different isolations. It is advisable to store some protein samples from isolations for which the results from Support Protocols 1 and 2 and the main assay in Basic Protocol 2 were satisfactory as a positive control in comparison of protein levels. Immunoblotting can also provide a parallel investigation to identify underlying causes of differences in ΔΨ_m,_ the OCR, or Ca^2+^ retention capacity. Together, Support Protocols 1 to 3 provide both quality‐control checks and additional assessment of mitochondrial physiology.

## Support Protocol 4 CRYOPRESERVATION OF ISOLATED MITOCHONDRIA

Because of the time needed and batch‐to‐batch variability, the mitochondria isolation procedure (Basic Protocol 1) can be the rate‐limiting step in high‐throughput assessment of mitochondrial permeability transition. Long‐term storage of isolated mitochondria is advisable to improve assay efficiency and consistency. Because freeze‐thaw cycles can disrupt both the OMM integrity of mitochondria and bioenergetic function, cryopreservation of mitochondria with intact OMM integrity and biological functions is accomplished using trehalose, a naturally occurring osmolyte (Yamaguchi et al., 2007). Using mitochondria isolated as described in Basic Protocol 1, execute the protocol described below to prepare tissue for long‐term storage and future use.

### Additional Materials (also see Basic Protocol 1)

Wash buffer (see recipe), 4°C
Homogenization buffer (see recipe) with cOmplete Protease Inhibitor, 4°C (Roche, 04693159001)
Liquid nitrogen

1 Place tissue (or cells; see Basic Protocol 1, step 1) in ice‐cold wash buffer in a 100‐ml glass beaker and mince with dissecting scissors.
2 Wash tissue with wash buffer three times to remove blood.
3 Drain wash buffer and replace it with ice‐cold homogenization buffer with cOmplete Protease Inhibitor. Homogenize using a tissue homogenizer as described in step 7 of Basic Protocol 1, once again using a volume in milliliters equaling twice the tissue's weight in grams.
4 Transfer homogenate to a 50‐ml Oak Ridge ultracentrifuge tube and centrifuge 10 min at 800 × *g*, 4°C, to remove the nuclear pellet.
5 Centrifuge supernatant 10 min at 10,300 × *g*, 4°C, to obtain the crude mitochondrial pellet.
6 Wash mitochondrial pellet by adding the same volume of homogenization buffer as in step 3 and repeating step 5.
7 Resuspend pellet in 1 ml homogenization buffer and determine protein concentration using a Pierce BCA Protein Assay Kit (see Basic Protocol 1, step 11).
8 Using liquid nitrogen, snap freeze mitochondrial suspension at a concentration of 50 mg protein/ml in homogenization buffer and store frozen samples at −80°C for ≤7 months (Briston et al., 2016).
  For subsequent use, thaw the mitochondria at 37°C in a water bath and maintain them on ice until functional analysis.

## INVESTIGATION OF CALCIUM RETENTION CAPACITY OF MITOCHONDRIA

### Basic Protocol 2 Investigation of Calcium Retention Capacity of Mitochondria as a Function of Amount of Ca^2+^ Buffered Until Permeability Transition

This protocol is designed to determine the capacity of the isolated mitochondria (Basic Protocol 1) to accumulate Ca^2+^ until the mPTP opens. Temporal fluorescence measurements of Ca^2+^‐sensing dyes in the extra‐mitochondrial buffer provide a simple and robust readout. An increase in fluorescence intensity in response to Ca^2+^ addition is closely followed by a decrease in fluorescence intensity as a result of an increase in mitochondrial Ca^2+^ uptake. Sequential additions of Ca^2+^ ultimately reach a threshold that results in opening of the mPTP, failure of the mitochondria to accumulate additional Ca^2+^, and Ca^2+^ release from the mitochondria. This results in a rapid increase in fluorescence intensity. This protocol uses a fluorescence plate reader with an integrated injector system for adding sequential Ca^2+^ pulses. For the purposes of drug or phenotypic screening, we recommend using CsA as a positive control for pore inhibition.

#### Materials

MSK buffer (see recipe)
1 M succinate (Sigma‐Aldrich, S3674)
1 mM rotenone (Sigma‐Aldrich, R8875) in DMSO (Sigma‐Aldrich, D8418)
1 mM Fluo‐5N (Invitrogen, 14203) in DMSO (Sigma‐Aldrich, D8418)
Calcium chloride (CaCl_2_)
190‐proof ethanol
Deionized water
Mitochondria (see Basic Protocol 1)

Fluorescence plate reader (Fluostar Optima, BMG Labtech), including integrated injector and pump
15‐ml Falcon tube (Fisher Scientific, 10263041)
96‐well clear plastic plate (VWR, 734‐0023)
Excel or equivalent software

**Figure 3:**
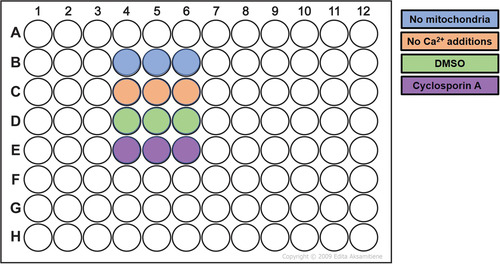
Assessment of the minimum conditions needed to determine the Ca^2+^ retention capacity of the isolated mitochondria, performed in triplicate. The mitochondria‐free condition provides a measure of the total Ca^2+^ added, and the Ca^2+^‐free addition helps define background fluctuations. Cyclosporin A is a cyclophilin D–dependent inhibitor of mitochondrial permeability transition pore opening, and dimethyl sulfoxide (DMSO) is the vehicle control for the cyclosporin A. A positive control for pore inhibition, such as cyclosporin A, is needed when using the assay for identifying drug candidates.

1 Set temperature of the fluorescence plate reader to 30°C.
2 Prepare MSK buffer supplemented with 10 mM succinate (stock concentration, 1 M) and 1 µM rotenone (stock concentration, 1 mM in DMSO) (MSK+) to energize the mitochondria and allow electrogenic mitochondrial Ca^2+^ uptake.
  We recommend using 20 ml MSK+ for a basic experiment, as described in Figure 3, and scaling up as needed for additional conditions.
  Succinate acts as a substrate for complex II, and rotenone inhibits complex I, thereby preventing reverse electron transfer through complex I and blocking accumulation of oxaloacetate, a known inhibitor of complex II activity.

**Figure 4:**
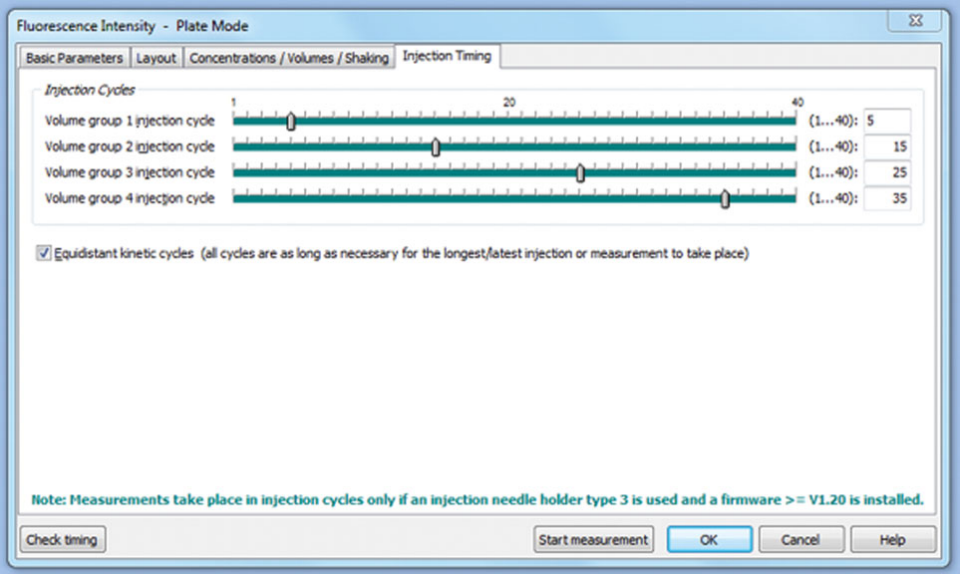
Protocol when using a BMG Labtech Fluostar with an injection every 10 cycles, beginning from cycle 5. This sequence is repeated three times, using the inbuilt script mode, to administer a total of 12 Ca^2+^ injections.

3 Add 1 mM Fluo‐5N (fluorescent dye) in DMSO to the MSK+ to a final concentration of 1 µM.
4 In a 15‐ml Falcon tube, prepare a 100 µM CaCl_2_ solution in the Fluo‐5N‐containing MSK+.
  A single 10‐µl sample of 100 µM CaCl_2_ is added beginning at cycle 5, and this is repeated every 10 cycles thereafter for a total of 12 additions (Fig. 4). This results in incremental accumulation of ∼10 µM Ca^2+^_._ Please note that the capacity of mitochondria to accumulate Ca^2+^ can vary based on the source of the mitochondria, that is, different tissues or cell lines, and the concentrations of the Ca^2+^ additions should be titrated accordingly to allow resolution of pore opening between controls and treatments that delay pore opening. This step can be performed in a pilot experiment, where ideally a concentration that causes pore opening between the third and ninth Ca^2+^ additions should be used.

5 Clean integrated injector of the plate reader with 190‐proof ethanol and deionized water before priming the pump with 1 ml of the CaCl_2_ solution.
6 Resuspend mitochondria in the MSK+ containing Fluo‐5N from step 3 to a final protein concentration of 500 µg/ml. Add 100 µl mitochondrial suspension to each well of a 96‐well clear plastic plate in triplicate for each condition (Fig. 3).
  A basic plate map for running the assay requires four conditions: wells without mitochondria, to which Ca^2+^ pulses are added, to represent the total Ca^2+^ added; wells with mitochondria but with no Ca^2+^ additions for background measurements; wells with mitochondria and Ca^2+^ additions to determine mPTP opening under control conditions; and finally, wells with mitochondria that have been pretreated with the positive control, CsA (10 mM in DMSO; Tocris, 1101), to isolate the effect of pore inhibition on Ca^2+^ retention capacity.
7 Analyze samples with the plate reader, adjusting the gain settings using a well containing only the CaCl_2_ solution as a maximum reading.
  The light source is a high‐energy xenon lamp. The emission from the excited sample is collected using a combination optic containing two liquid‐filled light guides for measuring fluorescence intensity. An excitation filter of 485 nm (bandpass: 12) is used, and the emitted light is detected with a side‐window photomultiplier tube after passing through a 520‐nm emission filter. Integrated injectors are used to perform the Ca^2+^ additions.
8 Analyze data using a simple spreadsheet in Excel or equivalent software, tracking the raw data for changes in fluorescence intensity over time. Given that the area under the curve for each condition represents the total Ca^2+^ buffered, calculate mitochondrial Ca^2+^ uptake as a fraction of the total Ca^2+^ (*‘buffering capacity’*) added:
1−∑i=1n condition −∑i=1n calcium  free ∑i=1n mitochondria  free
  Following this, perform comparisons to control untreated conditions by quantifying shift in the Ca^2+^ threshold for pore opening as a percentage change:
buffering  capacity − buffering  capacity  untreated  buffering  capacity  untreated ×100

### Multiplexing of Detection of Ca^2+^ Dynamics and Mitochondrial Swelling

Mitochondrial permeability transition is accompanied by mitochondrial swelling due to loss of IMM integrity. This can be measured as a decrease in light scattering. Historically, this technique was used to identify inhibitors of permeability transition. However, inhibition of mitochondrial Ca^2+^ uptake can also prevent mitochondrial swelling, resulting in false positives. Nevertheless, simultaneously measuring absorbance and Ca^2+^ retention capacity can help distinguish between inhibitors of mitochondrial Ca^2+^ uptake and inhibitors of pore opening.

**Figure 5:**
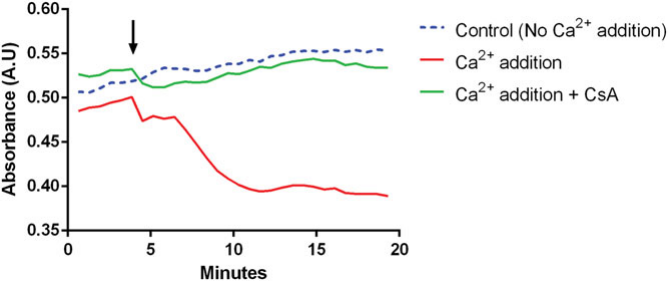
Typical absorbance curves for mitochondria isolated from mouse liver. In this example, a single bolus of Ca^2+^ is added (arrow) to trigger Ca^2+^‐induced pore opening. A loss of absorbance is observed compared to the condition with no Ca^2+^ addition. Cyclosporin A (CsA) can rescue this phenotype. Because the swelling assay lacks sensitivity compared to the Ca^2+^ retention capacity assay, a subtle difference in pore desensitization might be missed if using this assay alone.

The experimental setup is the same as that described in Basic Protocol 2 and does not require additional material. The additional measurement of absorbance is conducted simultaneously at 540 nm, and the samples would need to be set up in the plate as depicted in Basic Protocol 2. A decreased sensitivity to permeability transition is indicated by a reduced loss of absorbance compared to the control (Fig. 5). Pore opening in the Ca2+ retention capacity assay is mirrored by a steep negative gradient in the absorbance curve. This assay makes possible the measurement of two aspects of permeability transition: that is, mitochondrial swelling due to influx of water and unregulated mitochondrial Ca^2+ ^release. These data provide the user with additional confidence in the results with little extra effort in setting up the experiment.

## REAGENTS AND SOLUTIONS

All solutions should be prepared with deionized water, sterile filtered, and adjusted to pH 7.4. They should be prepared on the day of the experiment unless otherwise stated.

### Homogenization buffer

**Table:** 

Compound	Final concentration
Trehalose	300 mM
Potassium chloride	10 mM
EGTA	1 mM
EDTA	1 mM
HEPES	25 mM
Bovine serum albumin (BSA)	0.1% (w/v)

### Isolation buffer

**Table:** 

Compound	Final concentration
Mannitol	250 mM
EGTA	0.5 mM
HEPES	5 mM

### Miro5 buffer

**Table:** 

Compound	Final concentration
EGTA	0.5 mM
Magnesium chloride	3 mM
K‐lactobionate	60 mM
Potassium phosphate monobasic	10 mM
HEPES	20 mM
Sucrose	110 mM
BSA	0.001% (w/v)

Miro5 can be stored in 50‐ml aliquots for ≤3 months at −20°C. Once thawed, the pH should be adjusted to 7.4 at 37°C before use.

### MSK buffer

**Table:** 

Compound	Final concentration
Mannitol	75 mM
Sucrose	25 mM
Potassium phosphate monobasic	5 mM
Tris‐HCl	20 mM
Potassium chloride	100 mM
BSA	0.1% (w/v)

### Tris‐buffered saline

**Table:** 

Compound	Final concentration
Tris‐HCl	20 mM
Sodium chloride	150 mM

TBS can be stored ≤3 months at 4°C.

### Wash buffer

**Table:** 

Compound	Final concentration
Sucrose	250 mM
Potassium chloride	10 mM
EGTA	1 mM
EDTA	1 mM
HEPES	25 mM

## COMMENTARY

### Background Information

Mitochondria play a central role in [Ca^2+^]_c_ signaling. They accumulate Ca^2+^ in an electrogenic manner, mediated by the mitochondrial calcium uniporter complex (MCU) (Baughman et al., 2011). Increased [Ca^2+^]_m_ increases the activity of the three rate‐limiting enzymes of the tricarboxylic acid cycle, which in turn upregulates the production of NADH. This subsequently increases the rate of oxidative phosphorylation and ATP generation by providing reducing power for the electron transport chain (Griffiths & Rutter, 2009).

The mPTP was initially described in the 1970s by Haworth and Hunter as a high‐conductance pathway in the IMM that collapses ΔΨ_m_ (Haworth & Hunter, 1979; Hunter & Haworth, 1979; Hunter & Haworth, 1979). Numerous studies have demonstrated protection of tissues by CsA, most notably in the context of cardiac reperfusion injury (Andreeva, Tanveer, & Crompton, 1995; Duchen et al., 1998; Hausenloy, Duchen, & Yellon, 2003).

Two decades after the initial description of the phenomenon, cyclophilin D (CypD), a mitochondrial matrix protein, was identified as a modulator to pore opening. This was demonstrated convincingly by *in vivo* and *in vitro* knock‐out studies that resulted in mitochondria with an increased tolerance to Ca^2+^ loads (Andreeva & Crompton, 1994; Baines et al., 2005; Basso et al., 2005; Halestrap & Davidson, 1990; Nakagawa et al., 2005; Schinzel et al., 2005). Nonetheless, the central components of the pore have been the subject of much debate, with proteins found in both the OMM (VDAC, TSPO) and the IMM (ATP synthase, ANT, SPG7) proposed as candidates (Alavian et al., 2014; Beutner, Ruck, Riede, & Brdiczka, 1998; Bonora et al., 2017; Crompton, Virji, & Ward, 1998; Elustondo et al., 2016; Giorgio et al., 2017; Giorgio et al., 2013; Ricchelli, Sileikyte, & Bernardi, 2011; Ruck, Dolder, Wallimann, & Brdiczka, 1998; Shanmughapriya et al., 2015). Currently, there is significant uncertainty regarding the molecular composition of the pore (Baines, Kaiser, Sheiko, Craigen, & Molkentin, 2007; He, Carroll, Ding, Fearnley, & Walker, 2017; He, Ford, et al., 2017; Kokoszka et al., 2004; König et al., 2016; Sileikyte et al., 2014; Zhou, Marinelli, Nief, & Faraldo‐Gómez, 2017).

Although it has been suggested that transient opening of the pore occurs under physiological conditions, most studies have focused on its involvement in cell death (Korge et al., 2011). As a result of this work, the mPTP has been implicated in many disease pathologies associated with necrotic cell death, ischemia/reperfusion injury, neurodegenerative disorders such as Alzheimer's and Parkinson's diseases, and several forms of muscular dystrophy and myopathy (Devalaraja‐Narashimha, Diener, & Padanilam, 2009; Du et al., 2008; Dube et al., 2012; Hausenloy et al., 2003; Javadov et al., 2003; Millay et al., 2008; Palma et al., 2009; Thomas et al., 2012). The role of the mPTP has been most thoroughly documented in ischemia/reperfusion injury, particularly in tissues with a high metabolic demand, such as the brain, kidney, liver, and heart.

Although CypD is a known regulator of the mPTP and is therefore a druggable target, the uncertainty surrounding the molecular identity of the core components has been a significant hurdle in drug design and downstream validation. This has also impeded research into the involvement of the mPTP in disease and possible therapeutic interventions. Nevertheless, pharmacological targeting of elements thought to form the pore has provided positive results (for a review, see Briston et al., 2019).

As isolated mitochondria lack the physiological context of intact cells and tissues, functional validation of *in vitro* data in higher‐order systems is needed before selecting a clinical candidate. Cell‐based assays of the mPTP do not readily lend themselves to high‐throughput drug discovery programs. Among these assays is the calcein/cobalt technique, which can be used to probe pore opening at the level of the single cell. For this assay, cells are pre‐loaded with the fluorescent marker calcein‐AM. The dye distributes into all compartments, including the mitochondria, and is trapped after de‐esterification by nonspecific esterases. The cytosolic dye is then quenched by a short incubation with Co^2+^, which enters the cytosol but cannot enter intact mitochondria, resulting in calcein‐labeled mitochondria. Induction of mPTP opening by ionophores or oxidative stress results in quenching of the mitochondrial calcein signal as Co^2+^ enters the matrix through the now‐permeable IMM. The loss of ΔΨ_m_ following opening of the pore can also be measured using potentiometric cationic dyes such as TMRM, JC‐1, or rhodamine‐123. The methods used to induce pore opening can provide a false positive in these assays. For example, ionophores that transport Co^2+^ as well as Ca^2+^ across the IMM quench the mitochondrial calcein signal without opening the mPTP (Panel, Ghaleh, & Morin, 2017). An established inducer of the mPTP, such as ischemia/reperfusion, which provides more physiologically relevant induction of the pore by combining Ca^2+^ overload and oxidative stress, might be a better approach for intact cells. Therefore, the experimental design and interpretation of results are far more complex when assaying the mPTP in intact cells.

### Critical Parameters

#### Source of mitochondria

Mitochondria can be isolated (Basic Protocol 1) from virtually any tissue, including cultured cells. When designing the assay, if developing and testing a hypothesis that probes mitochondrial function within a tissue of interest, it is important to consider the physiological context. Other challenges, such as the ability to obtain an adequate yield of organelles and to access the tissue/cells of interest, influence the choice of isolation technique. The buffers employed as well as the instrument settings for differential centrifugation may be kept constant across different experimental setups. The ideal number of cells and/or amount of tissue needed to provide a sufficient yield must be optimized prior to initiating experiments. Furthermore, some tissue, such as muscle and heart, might require an additional protease lysis step prior to homogenization, whereas other tissues, such brain or cultured cells, require gentler homogenization. The functional integrity of the isolated mitochondria can be assessed by the quality‐control assays described in Support Protocols 1 to 3.

#### Ca^2+^‐sensing fluorescent dyes

Low‐affinity cell‐impermeant Ca^2+^ dyes are essential for correctly measuring the kinetics of mitochondrial Ca^2+^ uptake and mPTP opening (Basic Protocol 2 and Alternate Protocol). Because the Ca^2+^ retention capacity of mitochondria can vary across tissues, cell types, and isolation techniques, the most appropriate dye must be identified for the assay. Ideally, the K_d_ of the dye should be in the micromolar range. Shown in Table 1 are candidate Ca^2+^‐sensitive fluorescence indicators.

**Table 1:** Candidate Ca^2+^‐Sensitive Fluorescent Indicators

Dye	K_d_
Fluo‐5N	90 µM
Fluo‐4FF	9.7 µM
Calcium Green‐5N	14 µM
Fura‐FF^a^	6 µM

a Ratiometric indicators such as Fura‐FF provide a better quantitative measurement of Ca^2+^ changes while accounting for changes in dye loading and/or photobleaching.

#### Appropriate positive controls

It is crucial to include appropriate controls for assessing mitochondrial permeability transition in the assay design. CsA is the gold‐standard control (see Basic Protocol 2) for desensitizing the pore to inducers of pore opening, thereby delaying CypD‐dependent permeability transition. The contribution of CypD to pore opening can also be probed by genetic knock‐out or knock‐down. However, CypD inhibition only delays, and does not abolish, pore opening. The undefined nature of the pore complex currently makes it difficult to identify drug targets independent of CypD, although some exist, such as ER‐000444793 (Briston et al., 2016).

### Troubleshooting

The isolation technique (Basic Protocol 1) is often the source of technical errors encountered in executing these protocols. Overly harsh isolation conditions can damage the mitochondria and inhibit mitochondrial Ca^2+^ uptake. The quality‐control assays described in Support Protocols 1 to 3, and particularly retention of cytochrome c in the mitochondrial membrane (Support Protocol 3), are useful for identifying the optimum conditions for isolation of the mitochondria. Often, organelle isolation from different tissues results in a crude mitochondrial pellet that can be used in the assay of interest. However, contamination of the mitochondria with other tissue components (e.g., myelin in brain preparations) can negatively affect mitochondrial function. In such cases, a Percoll density gradient can be used to obtain enriched mitochondrial fractions with improved respiratory properties (Sims & Anderson, 2008).

### Understanding Results

**Figure 6:**
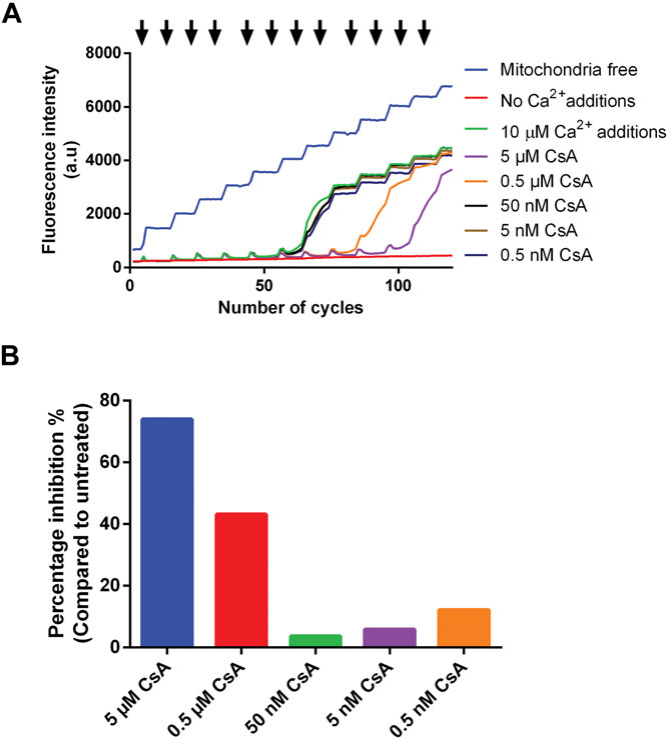
Concentration‐dependent response to cyclosporin A (CsA) in a Ca^2+^ retention capacity assay. (**A**) Samples of 10 µM CaCl_2_ (arrows) were added every tenth cycle after the initial addition at cycle 5 (arrows). An increase in fluorescence intensity after each addition is followed by a steep decline, indicating mitochondrial Ca^2+^ uptake. (**B**) Graphical representation of percentage inhibition as compared to untreated mitochondria.

A graphical representation of the raw data and quantification of pore opening in the presence of CsA are shown in Figures 6A and 6B, respectively. The data were analyzed using the method described in Basic Protocol 2, step 8.

### Time Considerations

The experimental workflow mentioned in Figure 1 requires ≥6 hr of time, which includes the time required for Basic Protocols 1 and 2. Basic Protocol 2 alone can take between 45 min and 3 hr, depending on the number of conditions per plate.

Support Protocols 1 and 2 do not need to be performed for every single isolation; however, these take 1 to 2 hr each when attempted.

It is critical that freshly isolated mitochondria are used for Support Protocols 1 and 2 and Basic Protocol 2, whereas mitochondria can be stored at −20°C for use in Support Protocol 3. The time required to complete Support Protocol 3 varies based on the system used for immunoblotting, ranging from 4 hr to 3 days with current technology.

The requirement to use freshly isolated mitochondria can be circumvented by isolating mitochondria according to Alternate Protocol, which allows the user to stop after protein quantification and proceed on a later date.

## References

[cpph59-bib-0001] Alavian, K. N. , Beutner, G. , Lazrove, E. , Sacchetti, S. , Park, H. A. , Licznerski, P. , … Jonas, E. A. (2014). An uncoupling channel within the c‐subunit ring of the F1FO ATP synthase is the mitochondrial permeability transition pore. Proceedings of the National Academy of Sciences of the United States of America, 111(29), 10580–10585. doi: 10.1073/pnas.1401591111. 24979777 PMC4115574

[cpph59-bib-0002] Andreeva, L. , & Crompton, M. (1994). An ADP‐sensitive cyclosporin‐A‐binding protein in rat liver mitochondria. European Journal of Biochemistry Febs, 221(1), 261–268. doi: 10.1111/j.1432-1033.1994.tb18737.x. 8168515

[cpph59-bib-0003] Andreeva, L. , Tanveer, A. , & Crompton, M. (1995). Evidence for the involvement of a membrane‐associated cyclosporin‐A‐binding protein in the Ca(2+)‐activated inner membrane pore of heart mitochondria. European Journal of Biochemistry Febs, 230(3), 1125–1132. doi: 10.1111/j.1432-1033.1995.tb20664.x. 7601144

[cpph59-bib-0004] Baines, C. P. , Kaiser, R. A. , Purcell, N. H. , Blair, N. S. , Osinska, H. , Hambleton, M. A. , … Molkentin, J. D. (2005). Loss of cyclophilin D reveals a critical role for mitochondrial permeability transition in cell death. Nature, 434(7033), 658–662. doi: 10.1038/nature03434. 15800627

[cpph59-bib-0005] Baines, C. P. , Kaiser, R. A. , Sheiko, T. , Craigen, W. J. , & Molkentin, J. D. (2007). Voltage‐dependent anion channels are dispensable for mitochondrial‐dependent cell death. Nature Cell Biology, 9(5), 550–555. http://www.nature.com/ncb/journal/v9/n5/supinfo/ncb1575_S1.html doi: 10.1038/ncb1575. 17417626 PMC2680246

[cpph59-bib-0006] Basso, E. , Fante, L. , Fowlkes, J. , Petronilli, V. , Forte, M. A. , & Bernardi, P. (2005). Properties of the permeability transition pore in mitochondria devoid of Cyclophilin D. Journal of Biological Chemistry, 280(19), 18558–18561. doi: 10.1074/jbc.C500089200. 15792954

[cpph59-bib-0007] Baughman, J. M. , Perocchi, F. , Girgis, H. S. , Plovanich, M. , Belcher‐Timme, C. A. , Sancak, Y. , … Mootha, V. K. (2011). Integrative genomics identifies MCU as an essential component of the mitochondrial calcium uniporter. Nature, 476(7360), 341–345. doi: 10.1038/nature10234. 21685886 PMC3486726

[cpph59-bib-0008] Beutner, G. , Ruck, A. , Riede, B. , & Brdiczka, D. (1998). Complexes between porin, hexokinase, mitochondrial creatine kinase and adenylate translocator display properties of the permeability transition pore. Implication for regulation of permeability transition by the kinases. Biochimica et Biophysica Acta, 1368(1), 7–18. doi: 10.1016/S0005-2736(97)00175-2. 9459579

[cpph59-bib-0009] Bonora, M. , Morganti, C. , Morciano, G. , Pedriali, G. , Lebiedzinska‐Arciszewska, M. , Aquila, G. , … Pinton, P. (2017). Mitochondrial permeability transition involves dissociation of F1FO ATP synthase dimers and C‐ring conformation. EMBO Reports, 18(7), 1077–1089. doi: 10.15252/embr.201643602. 28566520 PMC5494524

[cpph59-bib-0010] Briston, T. , Lewis, S. , Koglin, M. , Mistry, K. , Shen, Y. , Hartopp, N. , … Powney, B. (2016). Identification of ER‐000444793, a Cyclophilin D‐independent inhibitor of mitochondrial permeability transition, using a high‐throughput screen in cryopreserved mitochondria. Scientific Reports, 6, 37798. doi: 10.1038/srep37798. 27886240 PMC5122887

[cpph59-bib-0011] Briston, T. , Selwood, D. L. , Szabadkai, G. , & Duchen, M. R. (2019). Mitochondrial permeability transition: A molecular lesion with multiple drug targets. Trends in Pharmacological Sciences, 40(1), 50–70. doi: 10.1016/j.tips.2018.11.004. 30527591

[cpph59-bib-0012] Crompton, M. , Ellinger, H. , & Costi, A. (1988). Inhibition by cyclosporin A of a Ca2+‐dependent pore in heart mitochondria activated by inorganic phosphate and oxidative stress. Biochemical Journal, 255(1), 357–360. 3196322 PMC1135230

[cpph59-bib-0013] Crompton, M. , Virji, S. , & Ward, J. M. (1998). Cyclophilin‐D binds strongly to complexes of the voltage‐dependent anion channel and the adenine nucleotide translocase to form the permeability transition pore. European Journal of Biochemistry Febs, 258(2), 729–735. doi: 10.1046/j.1432-1327.1998.2580729.x. 9874241

[cpph59-bib-0014] Denton, R. M. , & McCormack, J. G. (1985). Ca2+ transport by mammalian mitochondria and its role in hormone action. American Journal of Physiology, 249(6 Pt 1), E543–554. 2417490 10.1152/ajpendo.1985.249.6.E543

[cpph59-bib-0015] Devalaraja‐Narashimha, K. , Diener, A. M. , & Padanilam, B. J. (2009). Cyclophilin D gene ablation protects mice from ischemic renal injury. American Journal of Physiology. Renal Physiology, 297(3), F749–759. doi: 10.1152/ajprenal.00239.2009. 19553348

[cpph59-bib-0016] Du, H. , Guo, L. , Fang, F. , Chen, D. , Sosunov, A. A. , McKhann, G. M. , … Yan, S. D. (2008). Cyclophilin D deficiency attenuates mitochondrial and neuronal perturbation and ameliorates learning and memory in Alzheimer's disease. Nature Medicine, 14(10), 1097–1105. doi: 10.1038/nm.1868. PMC2789841 18806802

[cpph59-bib-0017] Dube, H. , Selwood, D. , Malouitre, S. , Capano, M. , Simone, M. I. , & Crompton, M. (2012). A mitochondrial‐targeted cyclosporin A with high binding affinity for cyclophilin D yields improved cytoprotection of cardiomyocytes. Biochemical Journal, 441(Pt 3), 901–907. doi: 10.1042/BJ20111301. 22035570 PMC3260541

[cpph59-bib-0018] Duchen, M. R. (1992). Ca(2+)‐dependent changes in the mitochondrial energetics in single dissociated mouse sensory neurons. Biochemical Journal, 283(Pt 1), 41–50. doi: 10.1042/bj2830041. 1373604 PMC1130990

[cpph59-bib-0019] Duchen, M. R. , Leyssens, A. , & Crompton, M. (1998). Transient mitochondrial depolarizations reflect focal sarcoplasmic reticular calcium release in single rat cardiomyocytes. The Journal of Cell Biology, 142(4), 975–988. doi: 10.1083/jcb.142.4.975. 9722610 PMC2132882

[cpph59-bib-0020] Duchen, M. R. , Surin, A. , & Jacobson, J. (2003). Imaging mitochondrial function in intact cells. Methods in Enzymology, 361, 353–389. doi: 10.1016/S0076-6879(03)61019-0. 12624920

[cpph59-bib-0021] Elustondo, P. A. , Nichols, M. , Negoda, A. , Thirumaran, A. , Zakharian, E. , Robertson, G. S. , & Pavlov, E. V. (2016). Mitochondrial permeability transition pore induction is linked to formation of the complex of ATPase C‐subunit, polyhydroxybutyrate and inorganic polyphosphate. Cell Death Discovery, 2, 16070. doi: 10.1038/cddiscovery.2016.70. 27924223 PMC5137186

[cpph59-bib-0022] Giorgio, V. , Burchell, V. , Schiavone, M. , Bassot, C. , Minervini, G. , Petronilli, V. , … Bernardi, P. (2017). Ca2+ binding to F‐ATP synthase β subunit triggers the mitochondrial permeability transition. EMBO Reports, 18(7), 1065–1076. doi: 10.15252/embr.201643354. 28507163 PMC5494526

[cpph59-bib-0023] Giorgio, V. , von Stockum, S. , Antoniel, M. , Fabbro, A. , Fogolari, F. , Forte, M. , … Bernardi, P. (2013). Dimers of mitochondrial ATP synthase form the permeability transition pore. Proceedings of the National Academy of Sciences of the United States of America, 110(15), 5887–5892. doi: 10.1073/pnas.1217823110. 23530243 PMC3625323

[cpph59-bib-0024] Griffiths, E. J. , & Rutter, G. A. (2009). Mitochondrial calcium as a key regulator of mitochondrial ATP production in mammalian cells. Biochimica et Biophysica Acta (BBA) ‐ Bioenergetics, 1787(11), 1324–1333. doi: 10.1016/j.bbabio.2009.01.019. 19366607

[cpph59-bib-0025] Halestrap, A. P. , & Davidson, A. M. (1990). Inhibition of Ca2(+)‐induced large‐amplitude swelling of liver and heart mitochondria by cyclosporin is probably caused by the inhibitor binding to mitochondrial‐matrix peptidyl‐prolyl cis‐trans isomerase and preventing it interacting with the adenine nucleotide translocase. Biochemical Journal, 268(1), 153–160. doi: 10.1042/bj2680153. 2160810 PMC1131405

[cpph59-bib-0026] Hausenloy, D. J. , Duchen, M. R. , & Yellon, D. M. (2003). Inhibiting mitochondrial permeability transition pore opening at reperfusion protects against ischaemia–reperfusion injury. Cardiovascular Research, 60(3), 617–625. doi: 10.1016/j.cardiores.2003.09.025. 14659807

[cpph59-bib-0027] Haworth, R. A. , & Hunter, D. R. (1979). The Ca2+‐induced membrane transition in mitochondria: II. Nature of the Ca2+ trigger site. Archives of Biochemistry and Biophysics, 195(2), 460–467. doi: 10.1016/0003-9861(79)90372-2. 38751

[cpph59-bib-0028] He, J. , Carroll, J. , Ding, S. , Fearnley, I. M. , & Walker, J. E. (2017). Permeability transition in human mitochondria persists in the absence of peripheral stalk subunits of ATP synthase. Proceedings of the National Academy of Sciences of the United States of America, 114(34), 9086–9091. doi: 10.1073/pnas.1711201114. 28784775 PMC5576841

[cpph59-bib-0029] He, J. , Ford, H. C. , Carroll, J. , Ding, S. , Fearnley, I. M. , & Walker, J. E. (2017). Persistence of the mitochondrial permeability transition in the absence of subunit c of human ATP synthase. Proceedings of the National Academy of Sciences of the United States of America, 114(13), 3409–3414. doi: 10.1073/pnas.1702357114. 28289229 PMC5380099

[cpph59-bib-0030] Hunter, D. R. , & Haworth, R. A. (1979). The Ca2+‐induced membrane transition in mitochondria. III. Transitional Ca2+ release. Archives of Biochemistry and Biophysics, 195(2), 468–477. doi: 10.1016/0003-9861(79)90373-4. 112926

[cpph59-bib-0031] Hunter, D. R. , & Haworth, R. A. (1979). The Ca2+‐induced membrane transition in mitochondria: I. The protective mechanisms. Archives of Biochemistry and Biophysics, 195(2), 453–459. doi: 10.1016/0003-9861(79)90371-0. 383019

[cpph59-bib-0032] Javadov, S. A. , Clarke, S. , Das, M. , Griffiths, E. J. , Lim, K. H. H. , & Halestrap, A. P. (2003). Ischaemic preconditioning inhibits opening of mitochondrial permeability transition pores in the reperfused rat heart. The Journal of Physiology, 549(Pt 2), 513–524. doi: 10.1113/jphysiol.2003.034231. 12692185 PMC2342939

[cpph59-bib-0033] Kokoszka, J. E. , Waymire, K. G. , Levy, S. E. , Sligh, J. E. , Cai, J. , Jones, D. P. , … Wallace, D. C. (2004). The ADP/ATP translocator is not essential for the mitochondrial permeability transition pore. Nature, 427(6973), 461–465. doi: 10.1038/nature02229. 14749836 PMC3049806

[cpph59-bib-0034] König, T. , Tröder, S. E. , Bakka, K. , Korwitz, A. , Richter‐Dennerlein, R. , Lampe, P. A. , … Langer, T. (2016). The m‐AAA protease associated with neurodegeneration limits MCU activity in mitochondria. Molecular Cell, 64(1), 148–162. doi: 10.1016/j.molcel.2016.08.020. 27642048

[cpph59-bib-0035] Korge, P. , Yang, L. , Yang, J. H. , Wang, Y. , Qu, Z. , & Weiss, J. N. (2011). Protective role of transient pore openings in calcium handling by cardiac mitochondria. Journal of Biological Chemistry, 286(40), 34851–34857. doi: 10.1074/jbc.M111.239921. 21859717 PMC3186421

[cpph59-bib-0036] Lampl, T. , Crum, J. A. , Davis, T. A. , Milligan, C. , & Del Gaizo Moore, V. (2015). Isolation and functional analysis of mitochondria from cultured cells and mouse tissue. Journal of Visualized Experiments: JoVE, 0(97), 52076. doi: 10.3791/52076. PMC4401366 25866954

[cpph59-bib-0037] Millay, D. P. , Sargent, M. A. , Osinska, H. , Baines, C. P. , Barton, E. R. , Vuagniaux, G. , … Molkentin, J. D. (2008). Genetic and pharmacologic inhibition of mitochondrial‐dependent necrosis attenuates muscular dystrophy. Nature Medicine, 14(4), 442–447. doi: 10.1038/nm1736. PMC2655270 18345011

[cpph59-bib-0038] Nakagawa, T. , Shimizu, S. , Watanabe, T. , Yamaguchi, O. , Otsu, K. , Yamagata, H. , … Tsujimoto, Y. (2005). Cyclophilin D‐dependent mitochondrial permeability transition regulates some necrotic but not apoptotic cell death. Nature, 434(7033), 652–658. doi: 10.1038/nature03317. 15800626

[cpph59-bib-0039] Palma, E. , Tiepolo, T. , Angelin, A. , Sabatelli, P. , Maraldi, N. M. , Basso, E. , … Bonaldo, P. (2009). Genetic ablation of cyclophilin D rescues mitochondrial defects and prevents muscle apoptosis in collagen VI myopathic mice. Human Molecular Genetics, 18(11), 2024–2031. doi: 10.1093/hmg/ddp126. 19293339

[cpph59-bib-0040] Panel, M. , Ghaleh, B. , & Morin, D. (2017). Ca2+ ionophores are not suitable for inducing mPTP opening in murine isolated adult cardiac myocytes. Scientific Reports, 7(1), 4283. doi: 10.1038/s41598-017-04618-4. 28655872 PMC5487341

[cpph59-bib-0041] Prudent, J. , Popgeorgiev, N. , Gadet, R. , Deygas, M. , Rimokh, R. , & Gillet, G. (2016). Mitochondrial Ca2+ uptake controls actin cytoskeleton dynamics during cell migration. Scientific Reports, 6, 36570. https://www.nature.com/articles/srep36570#supplementary‐information. doi: 10.1038/srep36570. 27827394 PMC5101530

[cpph59-bib-0042] Ricchelli, F. , Sileikyte, J. , & Bernardi, P. (2011). Shedding light on the mitochondrial permeability transition. Biochimica et Biophysica Acta, 1807(5), 482–490. doi: 10.1016/j.bbabio.2011.02.012. 21377443

[cpph59-bib-0043] Ruck, A. , Dolder, M. , Wallimann, T. , & Brdiczka, D. (1998). Reconstituted adenine nucleotide translocase forms a channel for small molecules comparable to the mitochondrial permeability transition pore. Febs Letters, 426(1), 97–101. doi: 10.1016/S0014-5793(98)00317-2. 9598986

[cpph59-bib-0044] Schinzel, A. C. , Takeuchi, O. , Huang, Z. , Fisher, J. K. , Zhou, Z. , Rubens, J. , … Korsmeyer, S. J. (2005). Cyclophilin D is a component of mitochondrial permeability transition and mediates neuronal cell death after focal cerebral ischemia. Proceedings of the National Academy of Sciences of the United States of America, 102(34), 12005–12010. doi: 10.1073/pnas.0505294102. 16103352 PMC1189333

[cpph59-bib-0045] Shanmughapriya, S. , Rajan, S. , Hoffman, N. E. , Higgins, A. M. , Tomar, D. , Nemani, N. , … Madesh, M. (2015). SPG7 is an essential and conserved component of the mitochondrial permeability transition pore. Molecular Cell, 60(1), 47–62. doi: 10.1016/j.molcel.2015.08.009. 26387735 PMC4592475

[cpph59-bib-0046] Sileikyte, J. , Blachly‐Dyson, E. , Sewell, R. , Carpi, A. , Menabo, R. , Di Lisa, F. , … Forte, M. (2014). Regulation of the mitochondrial permeability transition pore by the outer membrane does not involve the peripheral benzodiazepine receptor (Translocator Protein of 18 kDa (TSPO)). Journal of Biological Chemistry, 289(20), 13769–13781. doi: 10.1074/jbc.M114.549634. 24692541 PMC4022851

[cpph59-bib-0047] Sims, N. R. , & Anderson, M. F. (2008). Isolation of mitochondria from rat brain using Percoll density gradient centrifugation. Nature Protocols, 3, 1228–1239. doi: 10.1038/nprot.2008.105. 18600228

[cpph59-bib-0048] Thomas, B. , Banerjee, R. , Starkova, N. N. , Zhang, S. F. , Calingasan, N. Y. , Yang, L. , … Starkov, A. (2012). Mitochondrial permeability transition pore component cyclophilin D distinguishes nigrostriatal dopaminergic death paradigms in the MPTP mouse model of Parkinson's disease. Antioxidants & Redox Signaling, 16(9), 855–868. doi: 10.1089/ars.2010.3849 21529244 PMC3292750

[cpph59-bib-0049] Yamaguchi, R. , Andreyev, A. , Murphy, A. N. , Perkins, G. A. , Ellisman, M. H. , & Newmeyer, D. D. (2007). Mitochondria frozen with trehalose retain a number of biological functions and preserve outer membrane integrity. Cell Death and Differentiation, 14(3), 616–624. doi: 10.1038/sj.cdd.4402035. 16977331

[cpph59-bib-0050] Zhou, W. , Marinelli, F. , Nief, C. , & Faraldo‐Gómez, J. D. (2017). Atomistic simulations indicate the c‐subunit ring of the F(1)F(o) ATP synthase is not the mitochondrial permeability transition pore. eLife, 6, e23781. doi: 10.7554/eLife.23781. 28186490 PMC5323039

